# Prenylated Flavonoids from *Cudrania tricuspidata* Suppress Lipopolysaccharide-Induced Neuroinflammatory Activities in BV2 Microglial Cells

**DOI:** 10.3390/ijms17020255

**Published:** 2016-02-19

**Authors:** Dong-Cheol Kim, Chi-Su Yoon, Tran Hong Quang, Wonmin Ko, Jong-Su Kim, Hyuncheol Oh, Youn-Chul Kim

**Affiliations:** 1 Institute of Pharmaceutical Research and Development, College of Pharmacy, Wonkwang University, Iksan 570–749, Korea; kimman07@hanmail.net (D.-C.K.); ycs1991@naver.com (C.-S.Y.); quangth2004@yahoo.com (T.H.Q.); rabis815@naver.com (W.K.); wpharm99@wku.ac.kr (J.-S.K.); hoh@wku.ac.kr (H.O.); 2 Institute of Marine Biochemistry, Vietnam Academy of Science and Technology (VAST), 18 Hoang Quoc Viet, Caugiay, Hanoi 10000, Vietnam

**Keywords:** *Cudrania tricuspidata*, cudraflavanone D, microglia, neuroinflammation, nuclear factor-κB, mitogen-activated protein kinase

## Abstract

In Korea and China, *Cudrania tricuspidata* Bureau (Moraceae) is an important traditional medicinal plant used to treat lumbago, hemoptysis, and contusions. The *C. tricuspidata* methanol extract suppressed both production of NO and PGE_2_ in BV2 microglial cells. Cudraflavanone D (**1**), isolated from this extract, remarkably suppressed the protein expression of inducible NO synthase and cyclooxygenase-2, and decreased the levels of NO and PGE_2_ in BV2 microglial cells exposed to lipopolysaccharide. Cudraflavanone D (**1**) also decreased IL-6, TNF-α, IL-12, and IL-1β production, blocked nuclear translocation of NF-κB heterodimers (p50 and p65) by interrupting the degradation and phosphorylation of inhibitor of IκB-α, and inhibited NF-κB binding. In addition, cudraflavanone D (**1**) suppressed the phosphorylation of c-Jun N-terminal kinase (JNK) and p38 MAPK pathways. This study indicated that cudraflavanone D (**1**) can be a potential drug candidate for the cure of neuroinflammation.

## 1. Introduction

*Cudrania tricuspidata*, a deciduous broadleaf thorny tree belonging to the Moraceae family, is spread throughout East Asia in Japan, Korea and China. According to Korean literature, this species has been used in oriental medicine for the treatment of poor health, impotency, and insomnia [1]. In addition, the root bark and cortex of this species have been used in traditional medicine for the therapy of inflammation and neuritis [2]. Previous phytochemical research has shown that *C. tricuspidata* contains several components such as xanthones [3], flavonoids [4], and glycoproteins [5]. In recent studies of the pharmacological effects of this plant, *C. tricuspidata* extracts have been shown to have various biological effects, including hepatoprotective [6], antioxidant [7], monoamine oxidase-A inhibitory [8], neuroprotective [9], anti-atherosclerotic, and anti-inflammatory activities [10].

Microglia have been traditionally defined as cerebral macrophages that may play important roles in neuroinflammation [11,12,13]. Moreover, microglia are a primary factor of the cerebral immune system [14]. When microglia are activated by a stimulus they produce neurotoxic mediators and pro-inflammatory cytokines including prostaglandin E_2_ (PGE_2_), nitric oxide (NO), interleukin-6 (IL-6), interleukin-1β (IL-1β), tumor necrosis factor-α (TNF-α), and superoxide anions [15,16]. In addition, microglial activation has been researched for its etiological role in neurodegenerative diseases (e.g., Alzheimer’s disease and ischemia *etc.*) [17,18]. Therefore, control of activated microglia would be an effective therapeutic approach for the range of neurodegenerative diseases.

Nuclear factor-κB (NF-κB) is a crucial factor in the responses of inflammation. NF-κB is activated by lipopolysaccharides (LPS) through the phosphorylation of inhibitor of kappa B-α (IκB-α). As a result, NF-κB translocates to the nucleus through separating from IκB-α [19,20]. Once NF-κB reaches the nucleus, this molecule binds to specific sites on DNA and regulates the transcription of its target genes; this leads to the production of pro-inflammatory mediators and cytokines such as cyclooxygenase-2 (COX-2), inducible NO synthase (iNOS), PGE_2_, NO, TNF-α, IL-6, and IL-1β [21,22].

Mitogen-activated protein kinases (MAPKs) consist of threonine-/serine-specific protein kinases, including extracellular signal-regulated kinase (ERK) 1/2 (p44/p42), c-Jun N-terminal kinase (JNK) and p38. These factors have important roles in cell differentiation, death, and proliferation [23] and are stimulated in signal transduction cascades by the phosphorylation of tyrosine/threonine residues.

In this study, the anti-inflammatory activity of *C. tricuspidata* was investigated as part of our wider efforts to discover medicinal plants which have anti-inflammatory effects. We found that seven prenylated flavonoids isolated from *C. tricuspidata* exerted anti-neuroinflammatory effects in LPS-induced microglial cells. Furthermore, the prenylated flavonoid cudraflavanone D (**1**) was selected to investigate the mechanism underlying these anti-neuroinflammatory activities, and the results suggested that it targeted COX-2 and iNOS expression through the MAPK and NF-κB signaling pathways.

## 2. Results

### 2.1. Structures of Compounds **1**–**7** and Cell Viability in BV2 Microglial Cells

**Figure 1:**
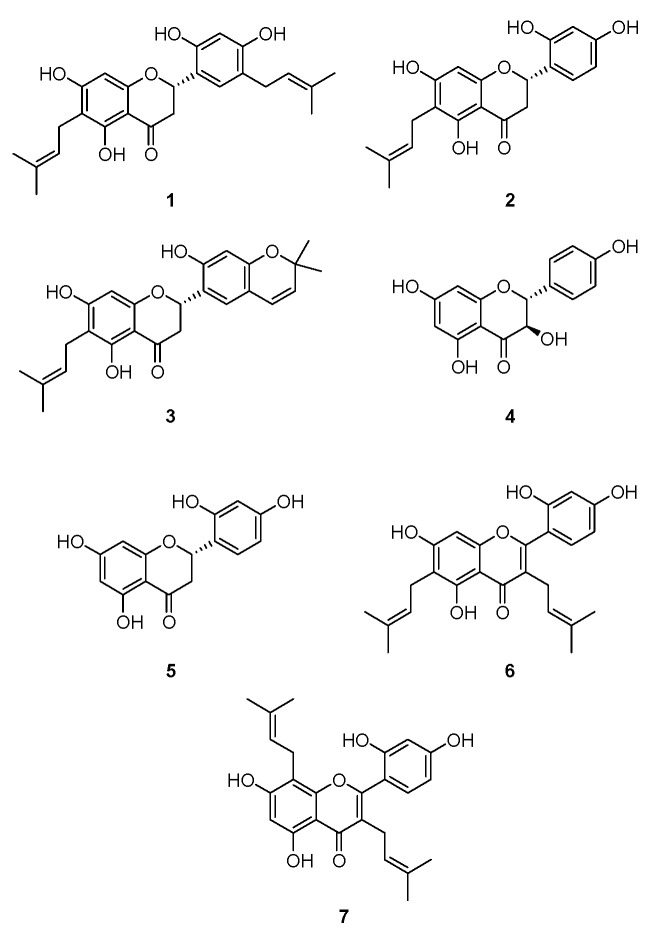
The structures of compounds **1**–**7**.

The structures of cudraflavanone D (**1**), cudraflavanone B (**2**), euchrestaflavanone C (**3**), (+)-dihydrokaempferol (**4**), steppogenin (**5**), cudraflavone C (**6**), and kuwanon C (**7**) (Figure 1) were identified in a previous study [24]. Before investigating the anti-neuroinflammatory potential of these compounds, the cytotoxicity of compounds **1**–**7** in BV2 microglial cells was estimated by using MTT assay. The individual compounds showed different cytotoxic effects and the non-toxic concentration range was determined for each compound (Figure S1). Non-toxic concentrations of compounds **1**–**7** were tested and are described below.

### 2.2. Effects of Compounds **1**–**7** on NO Production in LPS-Stimulated BV2 Microglial Cells

**Figure 2:**
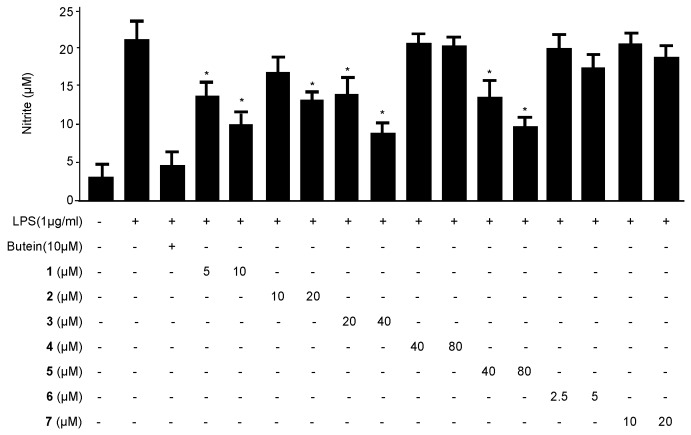
The effects of compounds **1**–**7** on nitrite production in BV2 microglial cells stimulated with LPS. The cells were pre-treated for 3 h with the indicated concentrations of compounds **1**–**7** and then stimulated for 24 h with LPS (1 μg/mL). The concentrations of nitrite were determined as described in the Materials and Methods section. The data represent the mean values ± SD of three experiments. * *p* < 0.05, as compared with cells treated with LPS only.

To determine the anti-neuroinflammatory effects of compounds **1**–**7** on LPS-induced BV2 microglial cells, the concentrations of the pro-inflammatory mediator NO were assessed in the absence and presence of non-cytotoxic concentrations of each compound. BV2 microglial cells were pretreated with the indicated compound for 3 h, followed by activation with LPS (1 µg/mL) for 24 h. In Figure 2, LPS treatment triggered an approximately eight-fold rise in the nitrite concentration of the culture media rather than the untreated cells. Compounds **1**–**3** and **5** inhibited the production of NO in a concentration-dependent manner, with IC_50_ values of 6.28 ± 0.31, 19.83 ± 0.99, 24.42 ± 1.22, and 43.55 ± 2.17, respectively. In contrast, compounds **4**, **6**, and **7** exhibited weak effects or no inhibitory effect at 80 µM. Butein was used as a positive control [25]. Following these findings, cudraflavanone D (**1**) had the strongest inhibitory effect and was selected for subsequent investigation of the underlying mechanism.

### 2.3. Effects of Cudraflavanone D (**1**) on TNF-α, IL-1β, IL-12, and IL-6 mRNA Expression in LPS-Stimulated BV2 Microglial Cells

**Figure 3:**
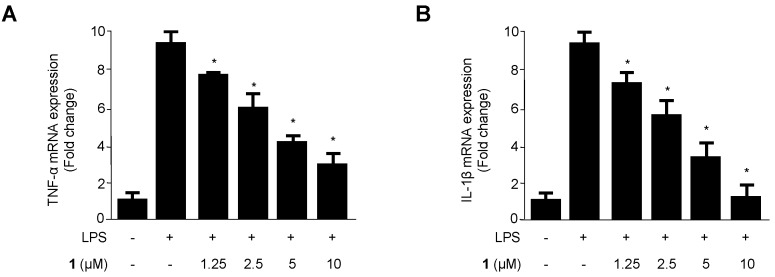

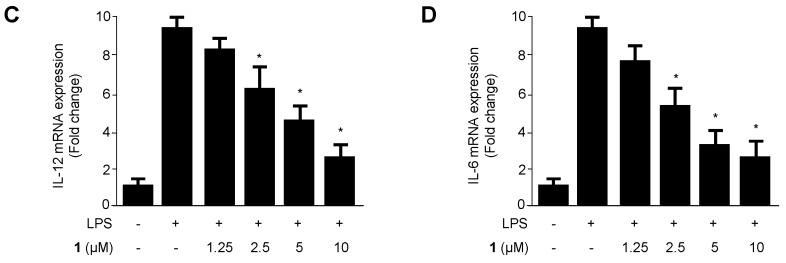
The effects of cudraflavanone D (**1**) on TNF-α (**A**), IL-1β (**B**), IL-12 (**C**), and IL-6 (**D**) mRNA expression in BV2 microglial cells stimulated with LPS. Cells were pre-treated for 3 h with the indicated concentrations of cudraflavanone D (**1**) and then stimulated for 12 h with LPS (1 μg/mL). The concentrations of TNF-α (**A**), IL-1β (**B**), IL-12 (**C**), and IL-6 (**D**) were determined as described in Materials and Methods. RNA quantification was performed as described in Materials and Methods and representative blots of three independent experiments are shown. The data represent the mean values of three experiments ± SD. * *p* < 0.05, as compared with the cells treated with LPS only.

We investigated the inhibitory effects of cudraflavanone D (**1**) on the production of pro-inflammatory cytokines (TNF-α, IL-1β, IL-12, and IL-6) in BV2 microglial cells. Cells were pre-treated with different doses of cudraflavanone D (**1**) induced by LPS for 12 h. As shown in Figure 3A–D, cudraflavanone D (**1**) decreased TNF-α, IL-1β, IL-12, and IL-6 production in a dose-dependent manner.

### 2.4. Effects of Cudraflavanone D (**1**) on PGE_2_ Production and iNOS and COX-2 Protein Expression in LPS-Stimulated BV2 Microglial Cells

**Figure 4:**
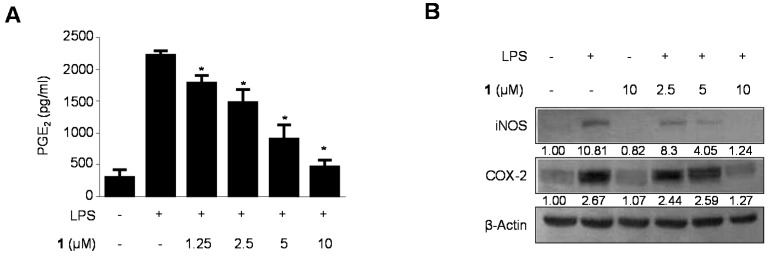
(**A**) The effects of cudraflavanone D (**1**) on protein expression of iNOS and COX-2 (**B**) in BV2 microglial cells stimulated with LPS. Cells were pre-treated for 3 h with the indicated concentrations of cudraflavanone D (**1**) and then stimulated for 24 h with LPS (1 μg/mL). The concentrations of iNOS and COX-2 (**B**) were determined as described in Materials and Methods. Western blot analyses were performed as described in Materials and Methods and representative blots of three independent experiments are shown. Band intensity was quantified by densitometry and normalized to β-actin; the values are presented below each band. Relative data represent the mean values of three experiments ± SD. * *p* < 0.05, as compared to the cells treated with LPS only.

We investigated the inhibitory effect of cudraflavanone D (**1**) on LPS-stimulated PGE_2_ production, and on iNOS and COX-2 protein expression (Figure 4). BV2 microglial cells were challenged with LPS (1 μg/mL) in the absence and presence of non-cytotoxic doses of cudraflavanone D (**1**) ranging from 1.25–10 µM. BV2 microglial cells pre-treated with cudraflavanone D (**1**) for 24 h resulted in decreased iNOS expression (Figure 4B) and reduced PGE_2_ production derived from COX-2 (Figure 4A). In the same conditions, cudraflavanone D (**1**) suppressed COX-2 expression (Figure 4B).

### 2.5. Effects of Cudraflavanone D (**1**) on IκB-α Levels, NF-κB Nuclear Translocation, and NF-κB DNA Binding Activity in LPS-Stimulated BV2 Microglial Cells

**Figure 5:**
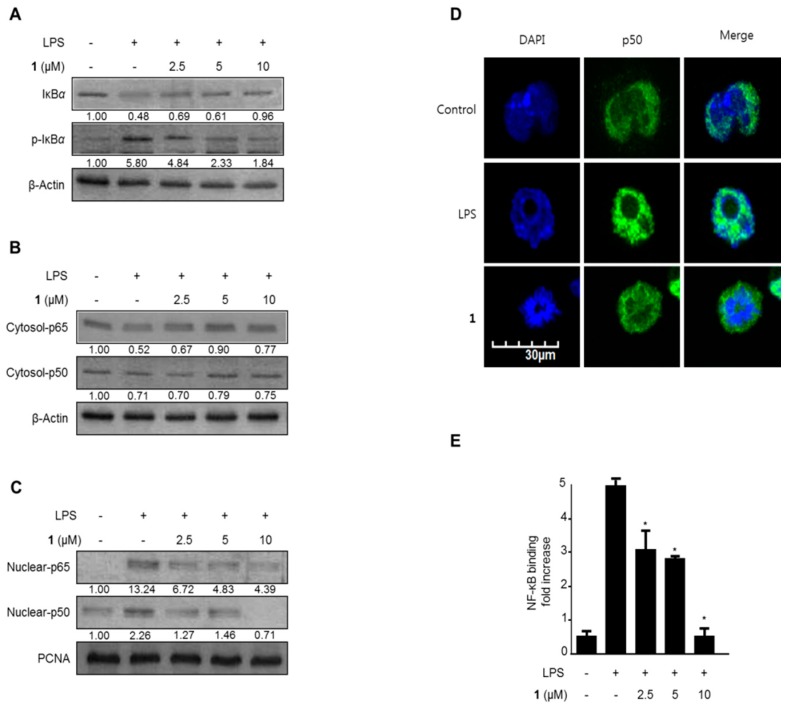
The effects of cudraflavanone D (**1**) on IκB-α phosphorylation and degradation (**A**), NF-κB activation (**B**,**C**), NF-κB localization (**D**), and NF-κB DNA binding activity (**E**) in BV2 microglial cells. Cells were pre-treated for 3 h with the indicated concentrations of cudraflavanone D (**1**), and then stimulated for 1 h with LPS (1 μg/mL). Western blot analyses of IκB-α and phosphorylated (p)-IκB-α in the cytoplasm (**A**), and NF-κB in the cytoplasm (**B**) and nucleus (**C**), and immunofluorescent analysis (**E**), were performed as described in Materials and Methods. Band intensity was quantified by densitometry and normalized to β-actin and PCNA, and the values are presented below each band. Relative data represent the mean values of three experiments ± SD. * *p* < 0.05, as compared to the cells treated with LPS only.

NF-κB activation stimulates expression of the iNOS and COX-2 proteins. NF-κB is inactive when bound to its inhibitor, IκB, in the cytoplasm. In response to external signals, NF-κB is separated from IκB and subsequently translocates to the nucleus [26,27]. Therefore, further investigation was conducted to determine whether cudraflavanone D (**1**) suppressed IκB-α degradation and phosphorylation, thus suppressing the NF-κB (p50 and p65) translocation to the nucleus. In Figure 5A, IκB-α was degraded after exposure of BV2 microglial cells to LPS for 1 h. However, pretreatment with 1.25–10 μM cudraflavanone D (**1**) for 3 h markedly suppressed this LPS-stimulated degradation and phosphorylation of IκB-α in a concentration-dependent manner, thus suppressing p50 and p65 nuclear translocation (Figure 5B,C). Furthermore, fluorescence microscopy identified that cudraflavanone D (**1**)-treated cells reduced NF-κB nuclear translocation as compared with untreated microglia (Figure 5D). We further experimented about NF-κB DNA binding activity in nuclear extracts from BV2 microglial cells activated by LPS for 1 h. This processing induced an approximately 10-fold rise in the DNA binding activity of NF-κB, which was inhibited by cudraflavanone D (**1**) in a concentration-dependent manner (Figure 5E).

### 2.6. Effects of Cudraflavanone D (**1**) on MAPK Phosphorylation in LPS-Stimulated BV2 Microglial Cells

**Figure 6:**
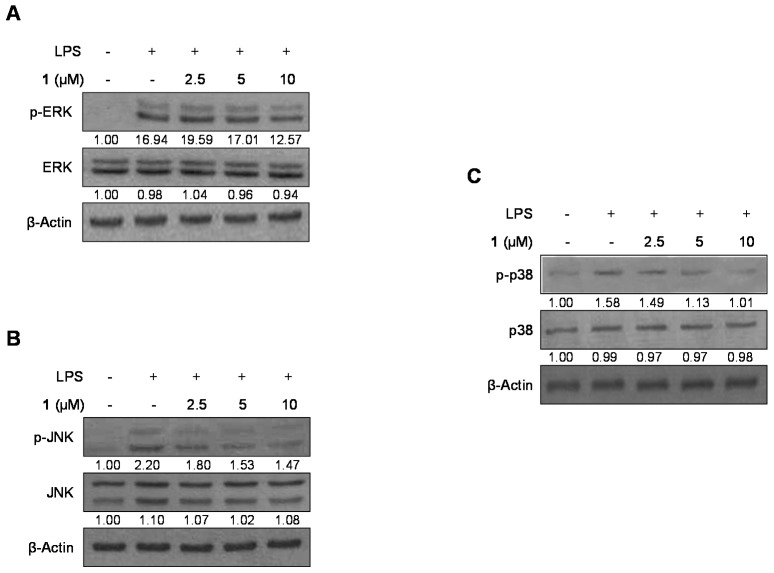
The effects of cudraflavanone D (**1**) on ERK, JNK, and p38 MAPK protein expression and phosphorylation. Cells were pre-treated for 3 h with the indicated concentrations of cudraflavanone D (**1**) and stimulated for 1 h with LPS (1 μg/mL) (**A**–**C**). The levels of (**A**) phosphorylated-ERK (p-ERK), (**B**) phosphorylated-JNK (p-JNK), and (**C**) phosphorylated-p38 MAPK (p-p38 MAPK) were determined by Western blotting. Representative blots from three independent experiments are shown. Band intensity was quantified by densitometry and normalized to β-actin; the values are presented below each band. Relative data represent the mean values of three experiments.

To investigate the MAPK pathway–mediated suppression of inflammation by cudraflavanone D (**1**), we assessed its effect on the LPS-stimulated phosphorylation of p38, JNK and ERK in BV2 microglial cells. In Figure 6, phosphorylation of p38, JNK and ERK was raised after being induced with LPS for 1 h. However, pre-treatment with 2.5–10 μM cudraflavanone D (**1**) for 3 h markedly suppressed the LPS-stimulated phosphorylation of JNK and p38 in a concentration-dependent manner (Figure 6B,C); however, ERK phosphorylation was not changed. Protein expressions of p38, JNK and ERK were unconverted by LPS. These data represented that cudraflavanone D (**1**)-mediated inflammatory responses by inhibiting the p38 and JNK MAPK signaling pathways.

## 3. Discussion

A literature survey revealed that several studies had reported the anti-inflammatory effects of *C. tricuspidata*. An ethyl acetate fraction of this plant was investigated to suppress the production of NO in LPS-induced RAW264.7 macrophages [28]. Similar effects were also observed using a chloroform fraction of this plant [29]. With respect to the components of the plant responsible for its effects on inflammation, a glycoprotein isolated from *C. tricuspidata* modulated activities of inflammatory signals in LPS-stimulated RAW264.7 cells [5]. Additionally, cudratricusxanthone A, a xanthone isolated from *C. tricuspidata*, inhibited inflammatory responses in LPS-stimulated RAW264.7 cells via effects on the expression of the heme oxygenase 1 enzyme [30]. Some flavonoids isolated from this plant were also reported to have anti-inflammatory effects. For example, Parks *et al.* reported that three flavonoids (cudraflavone B, cudraflavanone D and 2’,5,7-trihidroxy-4’,5’-(2,2-dimethylchromeno)-8-(3-hydroxy-3-methylbutyl)flavanone) produced anti-inflammatory effects by suppressing the protein expression of iNOS in LPS-stimulated RAW264.7 cells [10]. In addition, the anti-neuroinflammatory effect of methylalpinumisoflavone isolated from *C. tricuspidata* has been demonstrated to be mediated through the MAPK and NF-κB signaling pathways in LPS-induced microglial cells [31]. Cudraflavanone D (**1**) is a prenylated flavonoid from *C. Tricuspidata* that has been reported to have cytotoxic effects [32] and neuraminidase inhibitory activity [33]. In addition, an anti-inflammatory effect mediated via suppression of iNOS protein expression in LPS-stimulated RAW264.7 cells has been reported [10]. However, the anti-neuroinflammatory effects of cudraflavanone D (**1**) and other related flavonoids on microglia, and the molecular mechanism involved, are unknown. In this study, anti-neuroinflammatory effects of the prenylated flavonoids from *C. tricuspidata* on NO expression were identified in LPS-induced BV2 microglial cells. Therefore, further investigation was conducted to explore these effects in more detail, as well as the possible mechanisms involved.

Inflammation is an integrated response of an organism to various pathological changes, and it involves rapid upregulation and activation of many genes. This complicated process is modulated by pro-inflammatory mediators and cytokines, including NO, IL-1β, PGE_2,_ IL-6, IL-12 and TNF-α, in immune cells. The suppression of various mediators is critical during the treatment of inflammation. Recent researches have reported that inflammation is generated by iNOS and NO [34]. NO is synthesized by NOS, and inflammation is correlated with the level of iNOS [35]. Therefore, the suppression of iNOS and NO overproduction can be used to determine anti-inflammatory effects. We investigated whether prenylated flavonoids suppressed the production of NO during LPS-induced neuroinflammation in BV2 microglial cells. Some compounds had inhibitory effects on NO (Figure 2). PGE_2_, one of the inflammatory mediators, is generated at the inflammatory position by COX-2, an enzyme isoform that is stimulated in response to a variety of stimulants and that exacerbates inflammatory diseases [36,37]. Therefore, PGE_2_ and COX-2 are suggested as key enzymes for anti-inflammatory treatments. We investigated whether cudraflavanone D (**1**), a prenylated flavonoid component of *C. tricuspidata*, inhibited the expression of pro-inflammatory mediators, cytokines, and enzymes in LPS-stimulated inflammatory conditions in BV2 microglial cells. LPS stimulated COX-2 and iNOS expression in BV2 microglial cells, and these processes were suppressed by pre-treatment with cudraflavanone D (**1**) (Figure 5). Cudraflavanone D (**1**) also suppressed levels of the COX-2 product PGE_2_, and of the mRNA expression of the pro-inflammatory cytokines IL-12, IL-1β, IL-6, and TNF-α (Figure 2 and Figure 3).

LPS triggers its inflammatory effects by activating NF-κB, a transcription molecule that controls the expression of several genes such as IL-1β, COX-2, iNOS, and TNF-α. NF-κB heterodimers such as those composed of p65 and p50 are normally combined with IκB-α in the cytoplasm while NF-κB is regulated by various proteins such as TRAF6, TAK1, TRIF and CD14 which have a crucial role in translocation to the DNA binding site and phosphorylation of IκB [38]. Moreover, translocated NF-κB regulates the expression of pro-inflammatory proteins including TNF-α, COX-2, iNOS, IL-6, IL-12, and IL-1β, which are associated with neuroinflammation. [39]. However, in the existence of a pro-inflammatory stimulus, IκB-α is degraded and phosphorylated and NF-κB translocates to the nucleus, where it combines with target sites and induces the pro-inflammatory mediators [40]. We investigated the effects of cudraflavanone D (**1**) on IκB-α which degraded and phosphorylated, and on translocation of NF-κB heterodimers. After treatment with cudraflavanone D (**1**), LPS-stimulated IκB-α degradation and NF-κB activation were suppressed in BV2 microglial cells (Figure 5A–C). Furthermore, cudraflavanone D (**1**) decreased the NF-κB DNA binding activity (Figure 5E).

Many researchers have reported that MAPK intracellular signaling pathways are related to the regulation of inflammatory mediators [41,42]. We therefore investigated whether the anti-inflammatory effects of cudraflavanone D (**1**) in LPS-induced microglia involved the altered expression of MAPKs. Our results demonstrated that cudraflavanone D (**1**) was a strong inhibitor of the MAPK activation stimulated by LPS stimulation of BV2 microglial cells (Figure 6), indicating that these anti-inflammatory effects involved suppression of the MAPK signaling pathway.

In summary, prenylated flavonoid derivatives isolated from *C. tricuspidata* were identified to have inhibitory effects against NO expression in LPS-induced BV2 microglial cells. In the further study of the anti-neuroinflammatory effects of these metabolites, cudraflavanone D (**1**) was shown to suppress expression of LPS-stimulated COX-2 and iNOS at the protein level. In addition, **1** reduced the expression of pro-inflammatory cytokines. For the evaluation of the molecular mechanisms under the anti-inflammatory effects of **1**, the compound was found to suppress the NF-κB and MPAK signaling pathways in BV2 microglial cells stimulated with LPS. This regulation of the production of inflammatory molecules by cudraflavanone D (**1**) may have therapeutic potential against the range of neuroinflammatory and neurodegenerative diseases.

## 4. Experimental Section

### 4.1. Plant Materials

The root barks of *Cudrania tricuspidata* were purchased in May 2014 at Daerim Korean crude drug store, Kumsan, Chungnam Province, Korea, and identified by Dr. Kyu-Kwan Jang, Botanical Garden, Wonkwang University. A voucher specimen (No. WP-2014-12) was deposited at the Herbarium of the College of Pharmacy, Wonkwang University (Iksan, Korea). Prenylated flavonoids (Figure 1) were isolated from the methanol extract of *C. tricuspidata* (Moraceae) by various chromatographic methods, and the structures were determined mainly by analysis of MS and NMR data [24,43].

### 4.2. Chemicals and Reagents

Dulbecco’s modified Eagle’s medium (DMEM), fetal bovine serum (FBS), and other tissue culture reagents were purchased from Gibco BRL Co. (Grand Island, NY, USA). All other chemicals were obtained from Sigma Chemical Co. (St. Louis, MO, USA). Primary antibodies, including mouse/goat/rabbit anti-COX-2, iNOS, β-actin, IκB-α, phosphorylated (p)-IκB-α, p50, p65, proliferating cell nuclear antigen (PCNA), and secondary antibodies were purchased from Santa Cruz Biotechnology (Heidelberg, Germany), while p-ERK, ERK, p-JNK, JNK, p-p38, and p38 antibodies were obtained from Cell Signaling Technology (Cell Signaling, Danvers, MA, USA) [44].

### 4.3. Cell Culture and Viability Assay

BV2 microglial cells were received from Prof. Hyun Park at Wonkwang University (Iksan, Korea). BV2 microglial cells were maintained at 5 × 10^6^ cells/dish in 100-mm dishes in DMEM supplemented with 10% heat-inactivated FBS, penicillin G (100 units/mL), streptomycin (100 mg/mL), and l-glutamine (2 mM), and incubated at 37 °C in a humidified atmosphere containing 5% CO_2_ and 95% air. For the determination of cell viability, 2 × 10^4^ cells/well in 96-well plates were incubated with 3-(4,5-dimethylthiazol-2-yl)-2,5-diphenyltetrazolium bromide (MTT) at a final concentration of 0.5 mg/mL for 3 h, and the formazan formed was dissolved in acidic 2-propanol. Optical density was measured at 590 nm using a microplate reader (Bio-Rad, Hercules, CA, USA). The optical density of the formazan formed in control (untreated) cells was considered to represent 100% cell viability [43,44].

### 4.4. Quantitative Reverse-Transcription Polymerase Chain Reaction (qPCR)

Total RNA was isolated from the cells using Trizol (Invitrogen), in accordance with the manufacturer’s recommendations, and quantified spectrophotometrically at 260 nm. Total RNA (1 μg) was reverse transcribed using the High Capacity RNA-to-cDNA kit (Applied Biosystems, Carlsbad, CA, USA). The cDNA was then amplified using the SYBR Premix Ex Taq kit (TaKaRa Bio, Shiga, Japan) in a StepOnePlus Real-Time PCR system (Applied Biosystems). Briefly, each 20 μL reaction volume contained 10 μL SYBR Green PCR Master Mix, 0.8 μM of each primer, and diethyl pyrocarbonate-treated water. The primer sequences were designed using PrimerQuest (Integrated DNA Technologies, Cambridge, MA, USA). The primer sequences were: 5’-CCA GAC CCT CAC ACT CAC AA-3’ (forward) and 5’-ACA AGG TAC AAC CCA TCG GC-3’ (reverse) for TNF-α; 5’-AAT TGG TCA TAG CCC GCA CT-3’ (forward) and 5’-AAG CAA TGT GCT GGT GCT TC-3’ (reverse) for IL-1β; 5’-ACT TCA CAA GTC GGA GGC TT-3’ (forward) and 5’-TGC AAG TGC ATC ATC GTT GT-3’ (reverse) primers for IL-6; and 5’-AGT GAC ATG TGG AAT GGC GT-3’ (forward) and 5’-CAG TTC AAT GGG CAG GGT CT-3’ (reverse) for IL-12. The qPCR conditions were established by following the manufacturer’s instructions. The data were analyzed using StepOne software (Applied Biosystems) and the cycle numbers at the linear amplification threshold (*C*_t_) values for the endogenous control gene glyceraldehyde-3-phosphate dehydrogenase (GAPDH) and the target gene were recorded [43].

### 4.5. DNA Binding Activity of NF-κB

Microglia were pretreated for 3 h with the indicated concentrations of cudraflavanone D (**1**) and then stimulated for 1 h with LPS (1 μg/mL). The DNA-binding activity of NF-κB in nuclear extracts was measured using the TransAM kit (Active Motif, Carlsbad, CA, USA), according to the manufacturer’s instructions [45].

### 4.6. Preparation of Cytosolic and Nuclear Fractions

BV2 microglial cells were homogenized in PER-Mammalian Protein Extraction Buffer (1:20, *w*:*v*) (Pierce Biotechnology, Rockford, IL, USA) containing freshly added protease inhibitor cocktail I (EMD Biosciences, San Diego, CA, USA) and 1 mM phenylmethylsulfonylfluoride (PMSF). The cytosolic fraction of the cells was prepared by centrifugation at 16,000× *g* for 5 min at 4 °C. The nuclear and cytoplasmic cell extracts were prepared with NE-PER nuclear and cytoplasmic extraction reagents (Pierce Biotechnology, Rockford, IL, USA), respectively [44].

### 4.7. Nitrite Determination

The nitrite concentration in the medium, an indicator of NO production, was measured using the Griess reaction. Each supernatant (100 µL) was mixed with an equal volume of Griess reagent (Sigma; Solution A: 222488; Solution B: S438081), and the absorbance of the mixture at 525 nm was determined using a microplate reader [44].

### 4.8. Western Blot Analysis

BV2 microglial cells were harvested and pelleted by centrifugation at 16,000 rpm for 15 min. The cells were then washed with phosphate-buffered saline (PBS) and lysed with 20 mM Tris–HCl buffer (pH 7.4) containing a protease inhibitor mixture (0.1 mM PMSF, 5 mg/mL aprotinin, 5 mg/mL pepstatin A, and 1 mg/mL chymostatin). The protein concentration was determined using a Lowry protein assay kit (P5626; Sigma). An equal amount of protein from each sample was resolved using 7.5% and 12% sodium dodecyl sulfate-polyacrylamide gel electrophoresis and then electrophoretically transferred onto a Hybond enhanced chemiluminescence (ECL) nitrocellulose membrane (Bio-Rad, Hercules, CA, USA). The membrane was blocked with 5% skimmed milk and sequentially incubated with the appropriate primary antibody (Santa Cruz Biotechnology, CA, USA) and horseradish peroxidase-conjugated secondary antibody, followed by ECL detection (Amersham Pharmacia Biotech, Piscataway, NJ, USA) [43].

### 4.9. NF-κB Localization and Immunofluorescence

To study NF-κB localization, cells were grown on Lab-Tek II chamber slides and treated with 10 μM cudraflavanone D (**1**) for 0–60 min. Cells were then fixed in formalin and permeabilized with cold acetone. The cells were probed with p50 antibody followed by fluorescein isothiocyanate (FITC)-labeled secondary antibody (Alexa Fluor 488, Invitrogen). To visualize the nuclei, cells were then treated with 1 µg/mL 4’,6-diamidino-2-phenylindole (DAPI) for 30 min, washed with PBS for 5 min, and treated with 50 μL of VectaShield (Vector Laboratories, Burlingame, CA, USA). Stained cells were visualized using a Zeiss fluorescence microscope and photographed (Provis AX70, Olympus Optical Co., Tokyo, Japan) [46].

### 4.10. Statistical Analysis

The data are expressed as the mean ± standard deviation (SD) of at least three independent experiments. To compare three or more groups, one-way analysis of the variance was used, followed by Tukey’s multiple comparison tests. The statistical analysis was performed with GraphPad Prism software, version 3.03 (GraphPad Software Inc., San Diego, CA, USA) [46].

## 5. Conclusions

Treatment of BV2 microglial cells with cudraflavanone D (**1**) displayed a markedly dose-dependent suppression of lipopolysaccharide-induced NO production. In addition, cudraflavanone D (**1**) decreased the levels of pro-inflammatory cytokines. Cudraflavanone D (**1**) suppressed the expression of p38, and the JNK, MAPK, and NF-κB pathways in lipopolysaccharide-induced BV2 microglial cells. This study indicated that cudraflavanone D (**1**) represents a potential drug candidate for the treatment of neuroinflammation.

## Supplementary Materials

Supplementary materials can be found at http://www.mdpi.com/1422-0067/17/2/255/s1.
