# Supplementary material for: Prenylated Flavonoids from Cudrania tricuspidata Suppress Lipopolysaccharide-Induced Neuroinflammatory Activities in BV2 Microglial Cells
Source: Int J Mol Sci. 2016 Feb 19;17(2):255. doi: 10.3390/ijms17020255 (PMC4783984; doi:10.3390/ijms17020255)
Supplement: Supplementary file 1 [file ijms-17-00255-s001.pdf]

Supplementary Materials: Prenylated Flavonoids from *Cudrania tricuspidata* Suppress Lipopolysaccharide-Induced Neuroinflammatory Activities in BV2 Microglial Cells

Dong-Cheol Kim, Chi-Su Yoon, Tran Hong Quang, Wonmin Ko, Jong-Su Kim, Hyuncheol Oh and Youn-Chul Kim

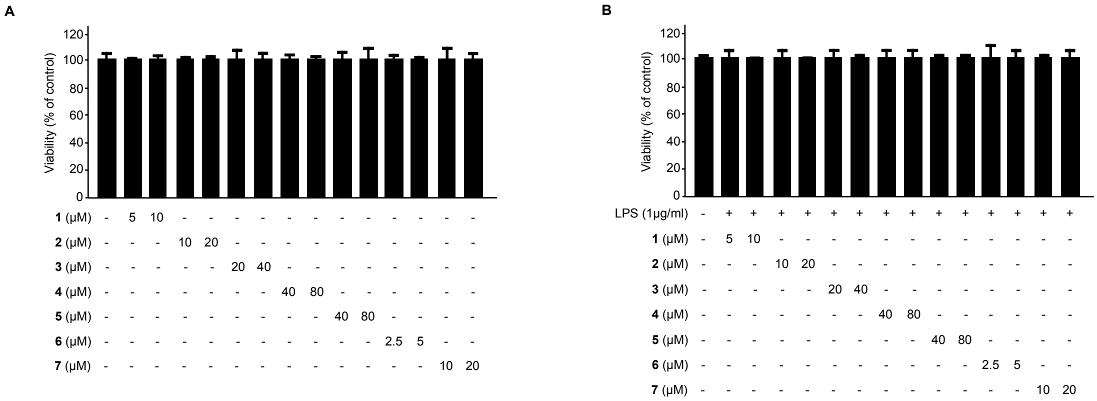

Figure S1. The viability effects of compounds **1**–**7** in BV2 microglia without LPS (**A**) and with LPS (**B**). BV2 microglia were incubated for 24 h with various concentrations of compound **1**–**7**. Cell viability was determined as described under Materials and Methods. The data represent the mean values ± SD of three experiments.
